# Protective Effect of Vitamin D Supplementation Against Atherosclerotic Cardiovascular Disease in Type 1 Diabetes Mellitus Model

**DOI:** 10.2174/0118715303341809241022110317

**Published:** 2025-01-13

**Authors:** Ayman Saeed Alhazmi

**Affiliations:** 1 Department of Clinical Laboratory Sciences, College of Applied Medical Sciences, Taif University, Taif, 24227, 20006, Saudi Arabia

**Keywords:** Vitamin D, cardiovascular disease, adropin, VEGF, CD4^+^, CD8^+^

## Abstract

**Introduction:**

Cardiovascular disease (CVD) is a leading cause of mortality on a global scale, with a higher prevalence observed among men. This study investigated the protective effect of vitamin D supplementation on CVD.

**Methods:**

A cohort of thirty mice was divided into three groups: control, T1 diabetic, and T1 diabetic groups that received vitamin D treatment. For each mouse in the three groups, measurements were taken of body weight, blood glucose levels, glycated hemoglobin (HbA1c), lipid profile, cardiac enzymes, troponin I, adropin, nitric oxide (NO), endothelin-1, and Vascular endothelial growth factor (VEGF). In addition, measurements were taken for the overall lymphocyte count, as well as the CD3^+^, CD4^+^, CD8^+^, CD4^+^, CD25^+^, and CD8^+^ CD25^+^ cell counts in all mice.

**Results:**

The diabetic mice that received vitamin D treatment exhibited significant reductions in blood glucose levels, HbA1c levels, lipid profile, cardiac enzymes, troponin I, endothelin-1, and VEGF levels as compared to the untreated diabetic group (*p* < 0.01). Furthermore, there was an observed rise in adropin and NO levels in diabetic mice that received vitamin D treatment compared to the untreated diabetic group (*p* < 0.05). The diabetic mice treated with vitamin D exhibited a substantial decrease in total lymphocyte counts compared to the untreated diabetic and control animals (*p* < 0.0001). Regarding the CD3^+^ subset, it was shown that diabetic mice subjected to vitamin D treatment had notably elevated levels of these cells compared to both the untreated diabetic and control groups (*p* < 0.0001). In addition, the administration of vitamin D resulted in a substantial decrease in the numbers of CD4^+^ and CD8^+^ cells in the group of individuals with diabetes (*p* < 0.0001). The diabetes group that received vitamin D treatment had significantly reduced populations of CD4^+^ CD25^+^ and CD8^+^ CD25^+^ compared to the untreated diabetic group (*p* < 0.0001).

**Conclusion:**

Vitamin D maintains the integrity of the cardiovascular system through the reduction of blood glucose levels and lipid profile. Moreover, its supplementation prevents atherosclerotic CVD by suppressing inflammatory reactions.

## 1 INTRODUCTION

Cardiovascular disease (CVD) kills most people worldwide, and men are more likely to have these incidents than women [1, 2]. Around 17.9 million people die from CVD Worldwide [3, 4]. New risk factors for CVD are emerging with potential prognostic and therapeutic implications in addition to familiar ones [5]. Recent research has focused on hypovitaminosis D [6, 7]. Lower vitamin D levels are common in 30-50% of the population, regardless of ethnicity or age [8, 9]. The discovery of a nuclear vitamin D receptor (VDR) in vascular endothelial cells and cardiomyocytes has revealed a direct role of vitamin D in CVD development and progression, prompting more studies on this topic [10, 11]. Vitamin D has been shown to have several cardiovascular effects, including anti-hypertrophic properties, inhibit cardiomyocyte proliferation, stimulate vascular smooth muscle cell proliferation, increase vascular endothelial growth factor (VEGF) expression, and inhibit renin-angiotensin-aldosterone system (RAAS) and natriuretic peptide secretion in experimental models [12, 13]. Observational studies implied that decreased 25(OH)D levels can affect cardiovascular health [14-17]. No consensus exists, however, on the appropriate vitamin level for CVD or cancer prevention; still, serum 25(OH)D levels > 30 ng/ml are vitamin D optimum [18-20]. Cardiovascular cells (vascular smooth muscle cells, endothelial cells, and cardiomyocytes), platelets, macrophages, dendritic cells, and other immune cells have VDR [13, 21, 22]. Age considerably reduces VDR expression regardless of serum 25(OH)D levels [23]. In cardiomyocytes, 1,25(OH)2D induces genomic and non-genomic actions that affect intracellular calcium metabolism [24-26]. In VDR knockout mice, renin and angiotensin II are increased, leading to hypertension and cardiac hypertrophy [27-30]. Cardiomyocyte-specific VDR deletion causes cardiac hypertrophy, whereas 1,25(OH)2D partially suppresses it in neonates [31, 32]. VDR deletion and vitamin D-deficient diets cause osteoblast-like cell development of vascular smooth muscle cells and aortic calcification [33-35]. Left ventricular hypertrophy, arterial stiffness, and vascular dysfunction can result from vitamin D insufficiency in humans. Moreover, left ventricle mass, atrial natriuretic peptide, homeostasis, cardiac metalloproteases, and fibroblasts increase without VDR, causing left ventricle dilation, fibrotic extracellular matrix development and improper electromechanical coupling [23, 36]. Growing evidence links immunological dysregulation and inflammation to several CVDs, such as atherosclerosis, myocardial infarction, arrhythmias, and heart failure. Recent research suggests that reducing inflammation may minimize cardiovascular events. Many CVDs, notably atherosclerosis, are linked to innate immunity. Neutrophils are the initial immune cells recruited to injured tissue and play a key role in local inflammatory process amplification. After the initial neutrophil burst, other immune cells, such as monocytes, macrophages, and lymphocytes, are drawn to injured tissue [37, 38]. Macrophages produce proinflammatory cytokines such as IFN-γ, IL-1β, IL-6, and IL-12. The role of the adaptive immune system has shown an association with CVD risk due to a sustained and chronic inflammatory state. T and B lymphocytes in atherosclerotic plaque may cause vascular inflammation through an autoimmune mechanism. Chronic inflammation has been linked to CVD risk by the adaptive immune system. T and B lymphocytes in atherosclerotic plaque may cause vascular inflammation through autoimmune mechanisms [39-41]. CD3^+^ T cells are recruited and primarily contribute to the maintenance of fibrous cap integrity. At the same time, CD4^+^ T cells also migrate to the site of inflammation. They can stimulate or suppress T-cells and tissue-resident cells, assist B-cells in producing antibodies, or display cytolytic activity, regulating the inflammatory environment at the plaque. CD8^+^ T cells promote atherosclerosis by controlling mouse monopoiesis and circulating monocyte levels [40, 42, 43]. Treatment with vitamin D reduced proinflammatory cytokines (IFN-γ and IL-17) while increasing anti-inflammatory cytokine (IL-10) production [44-46]. Vitamin D deficiency can cause inflammation in the arterial wall, leading to increased inflammation, atheroma formation, vascular smooth muscle cell proliferation, dysfunction, and inflammation [47-49]. The current study aimed to investigate the effect of vitamin D supplementation on cardiovascular integrity in diabetic mice.

## 2 MATERIALS AND METHODS

The present study was conducted in the Department of Applied Medical Sciences.

### 2.1 Experiment Design

Thirty male BALB/c mice, two months old, weighing 20-25 g, were purchased from Umm Al-Qura University's animal house. A conventional rodent enclosure with woodchip bedding was used to house the mice in a wide, well-aerated room with a 12-hour light and dark cycle at 25°C. All mice were fed rodent food and tap water during the study. The 30 mice were randomly assigned to three groups:

1. The negative control. No therapies were given to these mice.
2. The untreated diabetics. The mice in this cohort were given 55 mg/kg streptozotocin intraperitoneally for 5 days to produce T1DM.
3. The diabetic vitamin D-treated group. The mice were given 300 IU of vitamin D3 by intragastric route daily for six months after one week of the same streptozotocin therapy as the second group [50].

### 2.2 Measurement of Body Weight

Using a digital balance (OHAUS, Model: Scout Pro SPU601, China), the mice's body weights were measured every 6 weeks for six months.

### 2.3 Collection of Blood Samples

Blood samples were drawn blood from the retro-orbital venous plexus of all animals and collected in heparinized tubes for biochemical parameter estimation. Samples were immediately centrifuged at 2500 rpm for 15 min; then, the blood sera were kept at -80°C until analysis. Furthermore, blood samples were collected in an EDTA tube for flow cytometric analysis.

### 2.4 Estimation of Blood Glucose Levels and HbA1c

The random blood glucose levels were estimated using a colorimetric test before and after streptozotocin administration. Moreover, HbA1c was measured for all mice after 6 months with a glycohemoglobin kit (POINTE Scientific Inc., USA).

### 2.5 Estimation of Lipid Profile

Triglycerides, total cholesterol, HDL, and LDL were estimated for each mouse in all groups using colorimetric assays.

### 2.6 Estimation of Cardiac Enzymes

Serum AST, total CK, and CK-MB were estimated for each mouse in all groups using a colorimetric assay (kits purchased from the Beckman Coulter company).

### 2.7 Estimation of Cardiac Markers

Serum cardiac troponin I, adropin, nitric oxide (NO), ET-1, and VEGF were estimated using the quantitative competitive ELISA kits from the Thermo Fisher company. The serum levels of these parameters were measured using the Varioskan TM LUX instrument (Thermo Fisher Scientific, USA).

### 2.8 Estimation of Total Lymphocytes, CD3^+^, CD4^+^, CD8^+^, and CD25^+^ Subsets

The blood samples collected in the EDTA tubes were carefully placed on a Ficoll-Paque density gradient medium in a 15 mL conical tube. Samples underwent continuous 400×g centrifugation at 4°C for 30 min. The mononuclear cell layer was carefully removed and transferred to a fresh tube. The cells were rinsed twice with PBS and then stained with 2% fetal bovine serum and 0.1% sodium azide in PBS. The cell suspension was divided into four tubes, each with a 100 μL sample. Under stringent light protection, fluorochrome-conjugated monoclonal antibodies against CD4, CD8, CD25, and CD3 were incubated for 30 min at 4°C. The cells were rinsed with staining buffer and fixed with 1% paraformaldehyde for 30 min at 4°C under light protection. Subsequently, the samples were washed and reconstituted in the FACS buffer, and the samples were analyzed using a flow cytometer [51].

### 2.9 Statistical Analysis

SPSS 17 (SPSS Inc., Chicago, IL, USA) was used for statistical analysis. The data is presented as mean ± SD. The Comparison of body weight, blood glucose levels, HbA1c, triglyceride, total cholesterol, HDL, LDL, AST, total CK, CK-MB, cardiac troponin I, NO, adropin, ED-1, VEGF between all groups was made using one-way ANOVA. The significance level was set at *p* < 0.05. Flow cytometry data were analyzed using FlowJo software (Tree Star, Ashland, Oregon, USA).

## 3 RESULTS

### 3.1 Body Weight

Table **1** shows weight changes across all groups over six months. The body weight of untreated diabetic mice after six months significantly reduced compared to control groups (18.75 ± 2.18 *vs* 33.24 ± 2.08; *p* < 0.01). Diabetic mice treated with vitamin D had gained more weight than the untreated group after 6 months (30.66 ± 2.97 *vs* 18.75 ± 2.18; *p* < 0.01).

### 3.2 Blood Glucose Levels and HbA1c

The blood glucose levels for all groups 18 days after streptozotocin administration are shown in Table **2**. The blood glucose levels on days 6, 9,12,15, and 18 were significantly increased in untreated diabetic mice compared to the control group (*p* < 0.01). On days 15, 18, and 21, diabetic mice treated with vitamin D had reduced blood glucose levels compared to the untreated diabetic group (*p* < 0.01). Table **3** displays the percentages of HbA1c for all groups. HbA1c was significantly increased in untreated diabetic mice compared to the control group (13.10 ± 2.4 *vs* 5.20 ± 0.10; *p *< 0.01). Mice treated with vitamin D (6.78 ± 0.65) had significantly lower HbA1c levels than the untreated diabetic group (13.10 ± 2.4; *p* < 0.01).

### 3.3 Lipid Profile

Table **4** shows the lipid profiles of mice across all groups. Triglyceride, total cholesterol, HDL, and LDL levels were significantly increased in untreated diabetic mice compared with the control group (*p* < 0.01). Mice treated with vitamin D had considerably lower triglyceride levels (85.02 ± 4.61) than untreated diabetic mice (261.32 ± 8.04; *p* < 0.01). Moreover, the triglyceride level was significantly increased in untreated diabetic mice compared to the control group (261.32 ± 8.0 *vs* 491.33 ± 4.72; *p* < 0.01). Mice treated with vitamin D exhibited considerably lower total cholesterol levels (91.49 ± 5.67) than those in the untreated diabetic group (252.40 ± 5.31; *p* < 0.01). Diabetic mice treated with vitamin D had considerably lower LDL levels (53.31 ± 5.04) than the untreated diabetes group (211.28 ± 6.92; *p* < 0.01). Diabetic mice treated with vitamin D showed considerably greater HDL levels (63.42 ± 6.66) compared to the untreated diabetes group (20.00 ± 2.48; *p* < 0.01).

### 3.4 Cardiac Enzymes

The results of cardiac enzymes are shown in Table **5**. Untreated diabetic mice had considerably higher AST, total CK, and CK-MB levels than the control group (*p *< 0.01). Diabetic mice treated with vitamin D showed significantly lower AST activity (20.52 ± 3.88) than the untreated diabetic mice (93.61 ± 9.44; *p* < 0.05). The study found a significant decrease in total CK activity in diabetic mice treated with vitamin D (27.84 ± 2.06) compared to the untreated group (157.50 ± 24.19; *p* < 0.01). Moreover, diabetic mice treated with vitamin D had a significantly lower CK-MB (3.57 ± 0.58) than the untreated diabetic mice (32.11 ± 6.92; *p* < 0.01).

### 3.5 Cardiac Markers

The cardiovascular markers are shown in Table **6**. Positive cardiac integrity markers, adropin, and NO were significantly decreased in the untreated diabetic group compared with the control group ( *p* < 0.05). Furthermore, adropin was significantly upregulated in diabetic mice treated with vitamin D (27.10 ± 4.35) compared to the untreated group (7.80 ± 1.47) ( *p* < 0.05). Moreover, vitamin D significantly increased NO in diabetic mice (23.47 ± 2.01) compared to the untreated diabetic group (6.28 ± 1.49; *p* < 0.05). According to negative cardiac integrity markers, untreated diabetic mice had considerably higher ED-1 and VEGF compared to the control group ( *p* < 0.01). Vitamin D significantly reduced cardiac troponin I (33.04 ± 4.77) compared to untreated diabetic mice (167.21 ± 15.38; *p* < 0.01). Furthermore, vitamin D significantly decreased levels of cardiovascular dysfunction markers ET-1 and VEGF (12.44 ± 0.92 and 39.44 ± 5.41, respectively) compared to the untreated diabetic group (89.50 ± 11.61 and 278.49 ± 34.25, respectively; *p* < 0.01).

### 3.6 Peripheral CD3^+^, CD4^+^, CD8^+^, and CD25^+^ Subsets

Fig. (**1**) displays the total lymphocyte count of all groups. Total lymphocyte count was significantly increased in untreated diabetic mice compared to the control group ( *p* < 0.0001). Vitamin D treatment reduced the total lymphocyte count in diabetic mice compared to the untreated diabetic and control groups ( *p* < 0.0001). The CD3 ^+^ cell count of all groups is displayed in Fig. (**2**). The untreated diabetic mice had a higher CD3 ^+^ count than those in the control group ( *p* < 0.0001). Vitamin D therapy enhanced the numbers of CD3 ^+^ lymphocytes in diabetic mice compared with untreated diabetic and control groups ( *p* < 0.01 and 0.0001, respectively). Fig. (**3**) represents the CD4 ^+^ T-cell count of all groups. The CD4 ^+^ T-cell count was significantly higher in untreated diabetic mice compared to the control group (*p* < 0.0001). Moreover, Diabetic mice treated with vitamin D had significantly lower CD4 ^+^ T-cell numbers compared to the diabetic untreated and control groups ( *p* < 0.0001). Untreated diabetic mice had considerably higher CD8 ^+^ T-cell numbers than those in the control group (*p* < 0.0001; Fig. (**4** ). Mice in the diabetic group receiving vitamin D had lower CD8 ^+^ T-cell numbers than those in the untreated diabetic and control groups ( *p* < 0.0001; Fig. (**4** ). Fig. (**5**) displays CD4 ^+^ CD25 ^+^ T-cell count in all groups. The untreated diabetic group had higher CD4 ^+^ CD25 ^+^ T-cell numbers than the control group ( *p* < 0.0001). Vitamin D treatment significantly increased the number of CD4 ^+^ CD25 ^+^ T-cells in diabetic mice compared to the untreated and control groups ( *p* < 0.0001). According to CD8 ^+^ CD25 ^+^ T-cell, the untreated diabetic mice had higher numbers than the control group (*p* < 0.0001; Fig. (**6** ). Furthermore, diabetic mice who received vitamin D treatment had an increased number of CD8 ^+^ CD25 ^+^ T-cells compared to the untreated diabetic and control mice ( *p* < 0.0001; Fig. (**6**).

## 4 DISCUSSION

CVD is the main cause of morbidity and mortality worldwide. The most frequent CVDs are coronary heart disease, cardiac fibrillation, and heart failure [5]. Because the most common cause of CVD is diabetes mellitus, the study uses the diabetic-mouse model to investigate the correlation between CVD and vitamin D. Vitamin D supplementation and CVD study is ongoing due to inconsistent results [52, 53]. Bridie *et al.* concluded that vitamin D supplementation does not prevent CVD [54]. On the other hand, a follow-up study done on a large sample size for 10 years found that those with vitamin D deficiency risked acute myocardial infarction [55]. Vitamin D deficiency seems to be associated not only with CAD risk but also with its increased severity. In elective coronary angiography patients, vitamin D deficiency was linked to multi-vessel and diffuse CAD, as well as significant coronary artery stenosis [56]. Furthermore, a recent study showed that the supplementation of 60,000 IU of vitamin D3 per month for 5 years reduced major cardiovascular events, including myocardial infarction and coronary heart disease [57]. Evidence from population studies has shown a clear correlation between high total cholesterol and LDL levels that are involved in fatty streak formation and the risk of atherosclerosis. Moreover, low HDL levels augment this process [58]. The current study showed that vitamin D supplementation reduces triglyceride, total cholesterol, and LDL, whereas it increases HDL levels. Vitamin D may directly affect serum lipid profiles (triglyceride, total cholesterol, and LDL) by increasing the production of bile salts and reducing the activity of lecithin-cholesterol acyltransferase or indirectly by influencing calcium absorption, resulting in decreased fat absorption and increased synthesis of hepatic bile acids from cholesterol. Inflammation has become a significant factor in the development of atherosclerotic CVD and is also a crucial focus of treatment [59, 60]. Both innate and adaptive immunity are involved in the development of atherosclerotic plaque. T-cell-mediated inflammatory reactions have been recognized as a crucial factor in the development of CVD. In atherosclerotic plaque, naïve T- cells recognize antigenic peptides produced from lipids and endothelial cells presented by antigen-presenting cells (APCs). This process drives naïve T-cells to proliferate into CD4^+^ T-cells. CD4^+ ^T-cells are enriched in atherosclerotic plaque [61]. CD4^+^ T-cells further proliferate into T-helper cells (T_H1_ and T_H2_ cells). These cells release proinflammatory cytokines such as IFN-γ, IL-2, IL-17, and IL-4, so it is defined as a pro-atherosclerotic factor. Moreover, CD4^+ ^T-cells induce B-cell maturation, CD8^+^ T-cells, and macrophage activation. CD8^+^ T-cells are also enriched in atherosclerotic plaque. It plays a crucial role in monopoiesis and macrophage accumulation in early atherosclerosis. Furthermore, CD8^+ ^T-cells exert cytotoxic effects on atherosclerotic plaque by induction of macrophage cell death and the development of plaque necrotic core [62]. In atherosclerotic plaque, vitamin D produced by monocytes/macrophages leads to a significant change in immune function from a proinflammatory condition to a tolerogenic condition. Vitamin D inhibits the growth of CD4^+ ^T-cells into T_H1_ and T_H2_ and suppresses the production of proinflammatory cytokines (IL-2, IFN-γ, and IL-17) by these cells. Furthermore, a previous study conducted by Field *et al.* suggested that vitamin D decreases the absorption of LDL by macrophages by inhibiting the expression of scavenger receptors on the surface of macrophages. These findings were validated in individuals with different cardiovascular risk factors, such as diabetes mellitus [63]. A previous study reports a strong association between peripheral circulating CD4^+^ T-cell subsets and the occurrence of heart failure and atherosclerotic CVD [42]. Furthermore, Tanaka *et al.* suggest the existence of CD8^+^ T- cell subsets with different pathological functions in CVD [64]. The present study found that vitamin D supplementation reduces total lymphocytes in diabetic mice, whereas it increases naïve T-cells (CD3^+^). Moreover, vitamin D supplementation decreases both CD4^+^ and CD8^+^ T-cells in diabetic mice, which means that vitamin D plays a critical role in the prevention of inflammatory reactions that occur before the formation of atherosclerotic plaque by suppression the expansion of T-cells into CD4^+^ or CD8^+^ subsets. CD4^+^ CD25^+ ^T-cell, also known as T regulatory cell (Treg), is a lineage of T-cell that is derived from CD4^+^. These cells exert a protective effect and prevent atherosclerotic plaque development by modulating CD4^+^ and CD8^+^ T-cells and APCs, thereby inhibiting the production of pro-inflammatory cytokines. Moreover, Tregs produce anti-inflammatory cytokines, such as TGF-β and IL-10 [65]. Inhibition or dysfunction of Tregs might result in increased T_H1 _cells and APCs activities and enhance the production of pro-inflammatory cytokines, therefore inducing the inflammatory reaction [66]. PhospholipaseC-γ1 (PLC-γ1) is an important signaling protein participating in the classical T-cell receptor (TCR) signaling pathway. The key function of PLC-γ1 is to activate T cells and facilitate their proliferation into Tregs. Vitamin D induces Treg activation by increasing intracellular PLC-γ1 [67]. In the present study, vitamin D supplementation increases the numbers of CD4^+^ CD25^+^ T-cells (Tregs) subsets in diabetic mice treated with vitamin D compared with the untreated diabetic and control groups. The CD8^+^ CD25^+ ^T-cell, also known as CD8^+^ T regulatory cell (CD8^+ ^Treg) is a subset of T-cells. CD8^+^ Tregs have the same suppressive function as Tregs [68]. These cells decreased plaque size, reduced macrophage infiltration, and inhibited CD4^+^ T cell proliferation [61]. The current study showed that vitamin D supplementation induces CD8^+^ Tregs in diabetic mice. Interestingly, the results of cardiac enzymes and cardiac markers levels in plasma are corresponded with the T-cell subsets finding. The study indicated that supplementing diabetic mice with vitamin D lowered their cardiac enzymes (AST, total CK, and CK-MB) and troponin I, while those without treatment had elevated levels of these enzymes and troponin. Adropin is a protein composed of 43 amino acids produced by the liver, heart, lung, kidney, muscles, and blood [69]. Low adropin levels are linked to cardiovascular disease. Gallo *et al.* observed that adropin decrease predicts acute myocardial infarction in coronary artery disease patients [70]. Low blood adropin levels indicate coronary atherosclerosis in healthy and type 2 diabetes patients, so adropin is a predictor of a healthy cardiovascular system [71]. The present study found that vitamin D supplementation increased adropin levels in diabetic mice compared with diabetic untreated mice with high cardiac enzymes. NO is a second positive indicator of cardiovascular tissues. NO has anti-inflammatory, anti-oxidation, serum lipid level reduction, inhibition of vascular smooth muscle cell proliferation, and platelet aggregation activity. NO synthase dysfunction and low NO are implicated in CVD [72]. The current study showed that the diabetic mice have low NO levels. In the same study, vitamin D supplementation was shown to increase NO in diabetic mice. Endothelin-1 (negative cardiovascular integrity marker) is a 21 amino-acid peptide product of the *EDN1* gene. High endothelin-1 levels are risk factors for acute myocardial infarction. Several studies show that endothelin-1 is a predictor for post-myocardial infarction mortality [73]. The results of this study showed that vitamin D supplementation reduced endothelin-1 levels in diabetic mice. Vascular endothelial growth factor (VEGF) is a second negative cardiovascular integrity marker that increases in CVD. An investigation conducted on caucasian individuals with early-onset coronary artery disease indicates that high levels of circulating VEGF are linked to a higher risk of atherosclerosis [74]. VEGF stimulates the recruitment of inflammatory cells, mostly neutrophils and macrophages, and to a lesser degree, T-cells and B-immune cells [75]. Compared to untreated diabetic mice with high cardiac enzyme levels, vitamin D treatment reduced VEGF during the trial. Overall, vitamin D protects the cardiovascular system by suppressing inflammatory reactions that play a critical role in CVD.

## CONCLUSION

Vitamin D protects the cardiovascular system through the reduction of blood glucose levels and lipid profile. Moreover, vitamin D supplementation prevents atherosclerotic CVD by suppressing inflammatory reactions induced by immune response.

## STUDY LIMITATIONS

The research was specifically centered on the peripheral lymphocyte subsets only. Furthermore, it examines only a few subsets: CD4^+^, CD8^+^, and CD25^+^.

## Figures and Tables

**Fig. (1):**
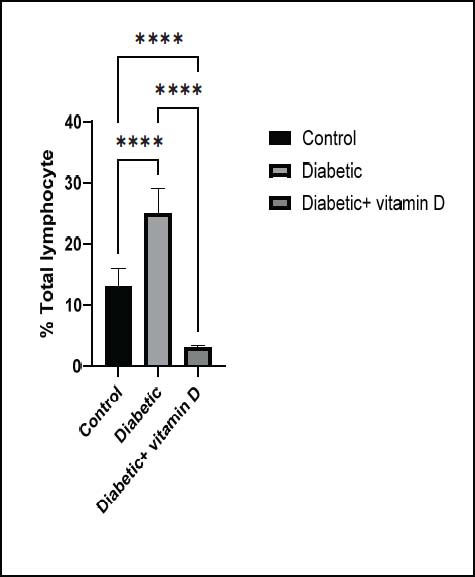
Total lymphocytes in all groups. Note: ** *p* > 0.01, **** *p* > 0.0001.

**Fig. (2):**
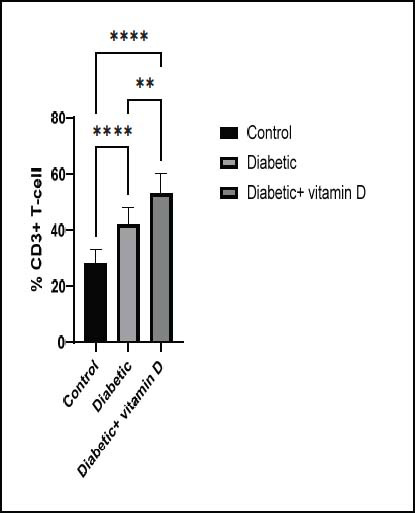
The CD^3+^ subsets in all groups. Note: ** *p* > 0.01, **** *p* > 0.0001.

**Fig. (3):**
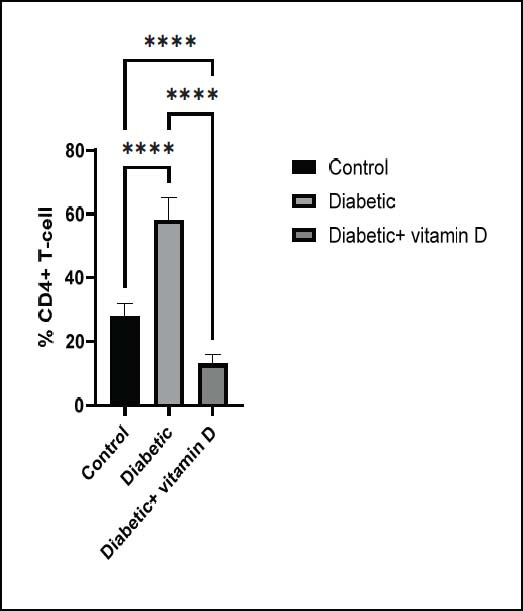
The CD^4+^ subsets in all groups. Note: ** *p* > 0.01, **** *p* > 0.0001.

**Fig. (4):**
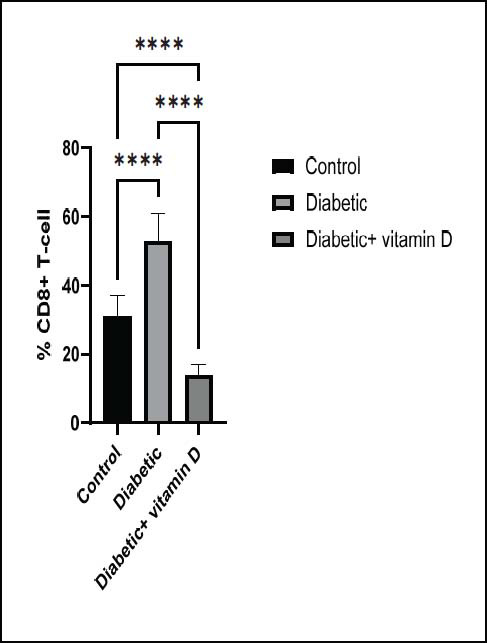
The CD^8+^ subsets in all groups. Note: ** *p* > 0.01, **** *p* > 0.0001.

**Fig. (5):**
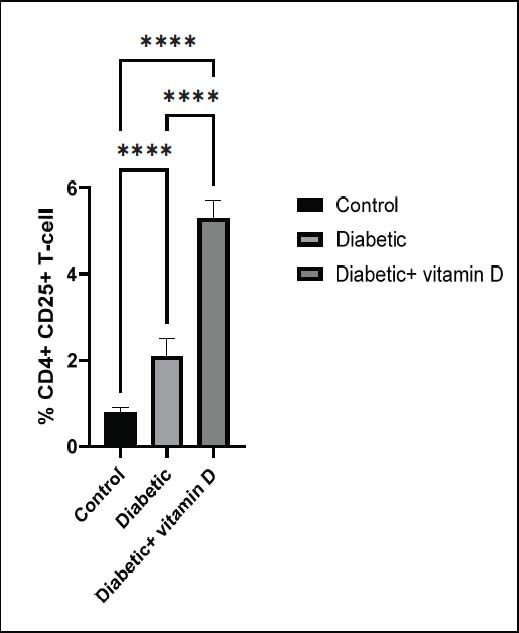
The CD^4+^ CD^25+^ subsets in all groups. Note: ** *p* > 0.01, **** *p* > 0.0001.

**Fig. (6):**
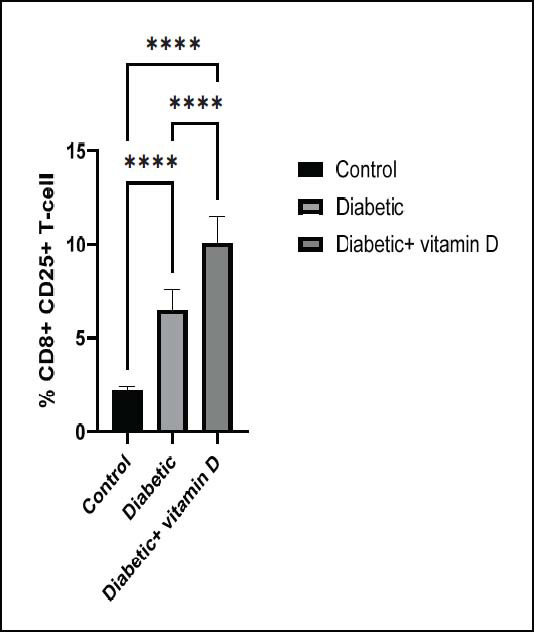
The CD^8+^ CD^25+^ subsets in all groups. Note: ** *p* > 0.01, **** *p* > 0.0001.

**Table 1:** Bodyweight of all groups.

-	** Control ** ** (n=10) **	** Diabetic ** ** (n=10) **	** Diabetic Treated with Vitamin D ** ** (n=10) **
** Body weight at beginning (g) **	23.36 ± 3.12	23.55 ± 2.91	23.55 ± 1.89
** After 6 weeks **	23.98 ± 3.17	23.61 ± 2.31	24.27 ± 2.03
** After 12 weeks **	24.25 ± 3.66	22.90 ± 3.58	25.55 ± 2.73
** After 18 weeks **	25.58 ± 2.77	22.02 ± 2.61	26.22 ± 1.38
** Body weight after six months (g) **	33.24 ± 2.08	18.75 ± 2.18	30.66 ± 2.97**

**Note:** ** *p* < 0.01 * *p* < 0.05.

**Table 2:** Blood glucose levels (mg/dL) of mice in all groups throughout five weeks.

-	Negative Control	Diabetic Group	Vitamin D Group
0 day	93.00 ± 7.00	91.00 ± 7.00	91.00 ± 8.00
6 days	91.00 ± 6.00	566.00 ± 33.00	434.00 ± 14.00
9 days	94.00 ± 5.00	465.00 ± 29.00	409.00 ± 21.00
12 days	92.00 ± 5.00	473.00 ± 20.00	366.00 ± 13.00
15 days	90.00 ± 4.00	411.00 ± 30.00	276.00 ± 12.00**
18 days	90.00 ± 6.00	455.00 ± 31.00	139.00 ± 27.00**
21 days	93.00 ± 4.00	463.00 ± 26.00	109.00 ± 8.00**

**Note:** * *p* < 0.05 ** *p* < 0.05.

**Table 3:** HbA1c of all groups after six months.

-	** Control ** ** (n=10) **	** Diabetic ** ** (n=10) **	** Diabetic Treated with Vitamin D ** ** (n=10) **
HbA1c %	5.20 ± 0.10	13.10 ± 2.4**	6.78 ± 0.65^1^**

**Note:** * *p* < 0.05 ** *p* < 0.05.

**Table 4:** Serum triglyceride, total cholesterol, LDL, and HDL of mice in six groups after six months.

-	** Control ** ** (n=10) **	** Diabetic ** ** (n=10) **	** Diabetic Treated with Vitamin D ** ** (n=10) **
** Triglyceride **	91.33 ± 4.72	261.32 ± 8.04	85.02 ± 4.61**
** t-cholesterol **	111.16 ± 6.42	252.40 ± 5.31	91.49 ± 5.67**
** LDL **	54.80 ± 4.51	211.28 ± 6.92	53.31 ± 5.04**
** HDL **	59.22 ± 3.62	20.00 ± 2.48	63.42 ± 6.66**

**Note:** ** *p* < 0.01 * *p* < 0.05.

**Table 5:** Cardiac enzymes for all groups.

-	** Control ** ** (n=10) **	** Diabetic ** ** (n=10) **	** Diabetic Treated with Vitamin D ** ** (n=10) **
** AST (IU/mL) **	24.55 ± 4.08	93.61 ± 9.44	20.52 ± 3.88*
** Total CK (IU/mL) **	20.33 ± 4.96	157.50 ± 24.19	27.84 ± 2.06**
** CK-MB (%) **	3.4 ± 0.96	32.11 ± 6.92	3.57 ± 0.58**

**Note:** ** *p* < 0.01 * *p* < 0.05.

**Table 6:** Cardiac markers for all groups.

-	** Control ** ** (n=10) **	** Diabetic ** ** (n=10) **	** Diabetic Treated with Vitamin D ** ** (n=10) **
** Cardiac troponin I **	23.4 ± 2.38	167.21 ± 15.38	33.04 ± 4.77**
** NO (pg/ml) **	22.04 ± 4.20	6.28 ± 1.49	23.47 ± 2.01*
** Adropin (µg/ml) **	18.42 ± 4.79	7.80 ± 1.47	27.10 ± 4.35*
** Endothelin (µg/ml) **	11.12 ± 3.33	89.50 ± 11.61	12.44 ± 0.92**
** VEGF (pg/mL) **	36.44 ± 6.53	278.49 ± 34.25	39.44 ± 5.41**

**Note:** ** *p* < 0.01 * *p* < 0.05.

## Data Availability

The datasets utilized and/or examined in the present work can be obtained from the relevant authors upon a reasonable request.

## LIST OF ABBREVIATIONS

APCs: Antigen-Presenting Cells
CVD: Cardiovascular Disease
NO: Nitric Oxide
RAAS: Renin-Angiotensin-Aldosterone System
VEGF: Vascular Endothelial Growth Factor
